# Analysis of the Distribution of Non-Metallic Inclusions and Its Impact on the Fatigue Strength Parameters of Carbon Steel Melted in an Electric Furnace

**DOI:** 10.3390/ma17246151

**Published:** 2024-12-16

**Authors:** Tomasz Lipiński

**Affiliations:** Faculty of Technical Sciences, University of Warmia and Mazury in Olsztyn, 10-719 Olsztyn, Poland; tomaszlipinski.tl@gmail.com

**Keywords:** structural steel, non-metallic inclusions, scatter index, fatigue resistance coefficient, fatigue strength

## Abstract

Steels are currently the most commonly used industrial construction materials. The use of steels depends on their properties, including their fatigue strength. Despite the fact that many works have been devoted to fatigue strength studies, there is still a lack of research discussing the fatigue strength of low-carbon steels. This deficiency is also visible when analyzing the influence of impurities on the fatigue properties of these steels. In most cases, the literature of material fatigue tests includes results obtained for materials produced on the laboratory scale, and it is difficult to directly translate these results to the industrial scale, on which steels for industrial applications are produced. This paper presents studies on the influence of non-metallic inclusions on the fatigue strength coefficient. The analyzed steel contained an average of 0.23% C, 1.23% Mn, and 0.0025 B. It was melted in 140-ton production furnaces, and after being tapped into a ladle, it was desulphurized and refined with argon. A classic plastic working process was used to produce steel samples. Based on the analysis of the test results, it was mainly found that the fatigue resistance coefficient k decreased with the increase in impurities spacing, and with a large share of smaller non-metallic inclusions, a higher fatigue resistance coefficient was noted, which may indicate that small non-metallic inclusions with an oval shape do not reduce the fatigue life of steel, regardless of its microstructure.

## 1. Introduction

Structural steels are the main material used to build the structural elements of machines and devices [1,2,3,4]. Designers’ requirements for steel depend on their working conditions. The group of steels subject to the highest requirements comprises those constantly operating under variable loads. The working conditions of these construction materials, in addition to their carefully selected chemical composition, require a homogeneous microstructure and high purity [5,6,7,8].

There are many known test results presenting the fatigue strength of steel. The data contained in these publications show that fatigue strength is a very complex process and depends on many factors [9,10,11,12]. The authors of these works show that fatigue life depends on the type of steel (chemical composition) and the manufacturing process, including treatments shaping the wet structure, which include heat plastic treatment. In the presented research results, the authors pay a lot of attention to the influence of impurities, especially non-metallic inclusions [13,14,15]. Non-metallic inclusions may enter the liquid steel from the outside, most often with the charge material, they may constitute defects in the furnace lining, or they may arise during the metallurgical process [16,17,18,19,20,21]. The literature classifies non-metallic inclusions into two groups [22,23]. The first group mainly includes particles originating from refractory materials, e.g., furnace linings, spouts, ladles, and other elements of the equipment of metallurgical stations. They enter the liquid steel from the outside and constitute exogenous pollutants. The second group includes chemical compounds formed in liquid steel during the metallurgical process, e.g., sulfides, oxides, and silicates. [24,25,26,27]. They are called endogenous impurities.

In the available publications, we can also find a mathematical model for loading stress and fatigue life that takes into account the hardness of steel, but it does not take into account the impurities present in it [28]. Researchers agree that large non-metallic inclusions cause a decrease in the fatigue strength of steel. Attempts have been made to link fatigue strength with the shape of impurities [29,30]. This analysis shows that the size and shape of impurities strongly determine the fatigue strength. Impurities with sharp edges cause destruction in the direction of the notch, while spheroidal impurities with small dimensions are not only able to move along grain boundaries but can cause multidirectional cracks. In addition to the above features, the authors indicate a significant role for the location of the impurities [31,32,33]. The numerous research results presented indicate the high complexity of the problem of the influence of non-metallic inclusions on the fatigue strength of steel.

Despite many studies having been conducted over several decades [34,35,36,37], the problem of the influence of non-metallic inclusions on the fatigue strength of steel has not been clearly explained, although many hypotheses have been presented. This confirms the relevance of this research topic. In the vast majority of studies, the authors present the impact of laboratory-introduced contaminants in liquid steel. For economic reasons, tests are carried out on melts conducted in laboratories in small furnaces. This approach to the topic does not explain the dependence of fatigue strength on inclusions. When conducting tests on steel with introduced impurities of a specific main fraction on a laboratory scale, it is possible to determine the fatigue strength of the tested material for the specific fraction of inclusions introduced. However, in industrial conditions, there are contaminants of various sizes, compositions, and morphologies. Therefore, results obtained in this way can only be used as a reference on the laboratory scale [38,39,40]. Also, research conducted in materials produced on a laboratory scale, starting with smelting and ending with heat treatment, has made it possible to produce an almost perfect material. This material may be characterized by high laboratory purity. It is also possible to perform almost perfect post-furnace and heat treatments that shape not only the microstructure [41] but also the quantity and quality of non-metallic inclusions. Steel produced in this way and its properties may differ significantly from the properties of steel produced in industrial conditions [42,43]. In a number of cases, in relation to materials produced in laboratory conditions, it is not possible to adapt the results obtained for use on an industrial scale. This happens not only in fatigue strength tests but also in many other tests conducted on the laboratory scale. This currently presents a key problem. This problem results from the economic conditions and the limited cooperation between research units and industry. Industrial plants, of course, have their own research teams that conduct research for their needs. However, they are not interested in making public the results of research that may indicate imperfections in the manufacturing methods used and, consequently, the products.

Secondary metallurgy and steel cleaning processes have a significant impact on the quantity and quality of non-metallic inclusions [44,45,46,47].

There are known relationships that allow the comparison of some properties of metal alloys. With high accuracy, for comparable steel grades, the fatigue strength of steel can be estimated at a known tensile strength *R_m_* (1) [48]. Of course, this relationship does not take into account differences in manufacturing processes, including the steel purity.

*z_g_* = *c* · *R_m_*, (1)

There are also well-known relationships that can be used to determine the static strength of the material based on its hardness (2) [48].

*R_m_* = *d* · *H*, (2)

Taking into account the dependencies in (1) and (2), it is possible to formulate a dependency that allows the estimation of the fatigue strength as a function of the steel hardness in the form of the fatigue resistance coefficient *k* (3).

(3) $$k=\frac{z_{g}}{H},$$

where

*z_g_*—fatigue strength, MPa;
*c*—the proportionality coefficient of fatigue strength and tensile strength;
*d*—the proportionality coefficient of tensile strength and hardness;
*R_m_*—tensile strength, MPa;
*H*—hardness, MPa;
*k*—fatigue resistance coefficient.

There are test results available in the literature for materials manufactured entirely on an industrial scale. Unfortunately, most of them are focused on explaining the causes of material damage or examining the fatigue properties of construction materials produced on an industrial scale, but the authors usually do not know the process of their production. Conclusions are made only on the basis of data obtained from the analysis of samples taken for testing. The results obtained in this way and the inferences made as a result should, in part, be considered intuitive in terms of reference to the manufacturing process.

Based on the above considerations, we decided to carry out tests on materials completely manufactured using a known industrial process. We decided to determine the chemical composition and dimension range of non-metallic inclusions and their impact on the fatigue strength coefficient of carbon steel melted in an electric furnace and subjected to desulfurization and argonization in a ladle.

## 2. Materials and Methods

For each series of samples from individual melts, the chemical composition was determined using an Applied Research Laboratories (ARL)—Quantometer 34000 by EVISA (Austin, TX, USA), a Leco analyzer CS744 (St. Joseph, MI, USA), and conventional analytical chemistry methods. The tests were carried out on structural steel with the chemical composition shown in Table 1.

The microstructure of the steel was analyzed using an OLYMPUS IX 70-S8F2 microscope (Japan) with OLYMPUS software DP-Soft. A scanning electron microscope Jeol JSM-7100F (Japan) with Jeol JSM 5400 equipped (Japan) with an ISIS 300 Oxford microprobe for microstructure and chemical composition analysis the non-metallic inclusions were used. The total volume of non-metallic inclusions was determined using chemical extraction. The dimensions of non-metallic inclusions were determined using a microscope at 400× magnification. Using this method, the volume of non-metallic inclusions was determined using size limits for the analyzed inclusions. Separate observation ranges were adopted for diameters greater than 2 μm, 5 μm, 10 μm, 15 μm, and 25 μm. The volume of non-metallic inclusions in individual dimensional ranges was determined analytically by subtracting the volumes determined for individual dimensional ranges. The volume of small non-metallic impurities with a diameter below 2 µm was calculated by subtracting the volume of inclusions larger than 2 µm determined using a video microscope from the volume of impurities determined by the chemical extraction method. The observation and counting of non-metallic inclusions using a video microscope were carried out on flat surfaces, assuming that the surface area of inclusions visible on the surface of the microsection was proportional to the volume of non-metallic inclusions present in the volume of the tested sample [49]. The video microscope was calibrated before each test separately for each of the tested dimensional ranges of impurities. PN-H-04510:1964 [50] was used for this purpose. The measurement error was checked before and after the tests. The error did not exceed 5% of the results obtained. The tested steel had a small content of impurities that comprised phosphorus and sulfur compounds, lower than the measurement error. For this reason, the focus was on the analysis of non-metallic oxide inclusions.

The tests were performed on 6 steel melts weighing 140 tons each. The melts were performed in a steelworks using an industrial arc furnace. The furnace charge in the form of pig iron was supplemented with 25% steel scrap. After melting, the steel was poured into ladles holding seven tons of alloy each. The desulphurization process was carried out in the ladle. Next, the steel was poured into the ladle and refined with argon. Argon was introduced into the liquid steel through a porous brick. The argon purging time ranged from 8 to 10 min.

After desulphurization and argon refining, the ingots were rolled into bars measuring 100 × 100 mm. From each melt, 51 samples were taken, and fatigue strength tests were performed during rotational bending. Samples for static tensile test and fatigue strength tests were prepared, ensuring that the direction of the sample axis was parallel to the direction of rolling the bars (parallel grain distribution in the samples).

In industry, steels are used for elements operating in various different conditions and under various different loads. Therefore, steels with different mechanical properties are needed. Studies have shown that the mechanical properties of steel, including fatigue life, are closely correlated with its microstructure. The tensile strength and hardness of steel are also a result of the microstructure. In this study, we decided to test steel with different microstructures and thus with different properties. To achieve this goal, the steel was subjected to heat treatment. After preparation, the samples were austenitized at a temperature of 880 °C for 20 min, after which they were cooled in water. To ensure a diverse microstructure, tempering was applied to individual batches of samples at different temperatures, from 200 °C to 600 °C, changing every 100 °C. The heating time was 120 min, with air cooling. The heat treatment was carried out in an electric furnace LH 15/14 manufactured by Naberterm GmbH /lilinthal. The static tensile test was carried out in accordance with PN-EN ISO 6892-1:2020-05 [51] on five-fold samples with a diameter of 8 mm by ZD 30 VEB Wergzeugmaschinenkombinat “Fritz Heckert” Karl-Marx-Stadt (Germany). The hardness was measured using the Vickers method in accordance with PN-EN ISO 6507-1:2024-04 [52] by HPO 250 VEB Werkstoff Pruf Maschinen Leipzig by Wergzeugmaschinenkombinat “Fritz Heckert” (Germany). To determine the fatigue strength, rotational bending with a frequency of 6000 cycles per minute was used [53], selecting the load during the test for the average static strength of the samples, which was determined separately for each heat treatment variant. The fatigue tests were carried out on a VEB Werkstoff Pruf Maschinen Leipzig rotary bending machine manufactured by Wergzeugmaschinenkombinat “Fritz Heckert” (Germany) with Thermo-hunter BA-06TA OPTEX CO. LTD (Otsu, Japan). The stress value was determined experimentally, assuming a durability of 10^7^ cycles (Table 2).

The scatter index, which is the quotient of the average pollution size and the average distance between the pollution, was determined from the relationship (4).

(4) $$\chi =\frac{\lambda }{\bar{d}},$$

where

(5) $$\bar{\lambda }=\frac{2}{3}\bar{d}\left(\frac{1}{V}-1\right),$$

$\bar{d}$—average diameter of impurities, µm,
*V*—relative volume of impurities, %.

The steel fatigue resistance coefficient k for each and every tempering temperature used can be presented in a general form, as seen in Equation (6):

*k*_(*tempering temp.*)_ = *a* · χ + *b*, (6)

where

*k_(tempering temp.)_*—fatigue resistance coefficient for the assumed tempering temperature;
*a* and *b*—coefficients of the equation;
χ—scatter index.

The significance of the correlation coefficient r of each of the regression equations was determined based on Student’s t-test statistical distribution for the significance level α = 0.05 and the number of degrees of freedom f = n − 1.

The dissipation fatigue resistance coefficient *δ_(tempering temperature)_* for each of the regression equations *k_(tempering temperature)_* (6) was calculated by (7), as follows:

(7) $$\delta_{\left(tempering\;temp.\right)}=\frac{2\cdot s}{\sqrt{1-r^{2}}}$$

where

*s*—standard deviation;
*r*—correlation coefficient.

## 3. Results

**Figure 1:**
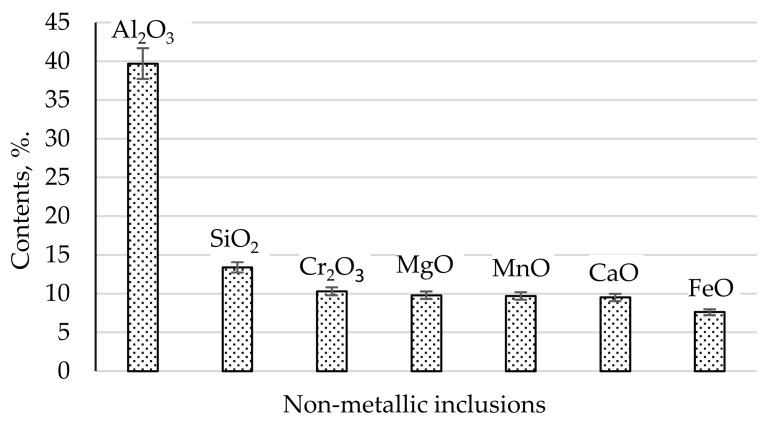
Chemical structure of oxide impurities contents in the tested steel.

The content of non-metallic inclusions in the analyzed steel determined on the basis of chemical extraction tests was 0.18% of the steel volume. An example of chemical structure non-metallic inclusions occurring in the analyzed steel is shown in Figure 1 (assuming that the sum of all impurities is 100%).

The largest volume of non-metallic inclusions of all impurities was noted for Al_2_O_3_, at close to 40%. Non-metallic SiO_2_ inclusions constituted about 14%, while Cr_2_O_3_ comprised about 10%. The volume fractions of the phases MgO, MnO, and CaO were determined at a similar level, slightly below 10%. FeO represented the lowest content, the volume of which was determined to be below 8%. Therefore, the dominant non-metallic inclusion in terms of the volume occupied was Al_2_O_3_, which, together with SiO_2_, occupied the majority volume of about 53% in the microstructure of the analyzed steel. All analyzed non-metallic inclusions were oxides. Non-metallic inclusions that were not oxides may have occurred in the steel below the level of determination.

**Figure 2:**
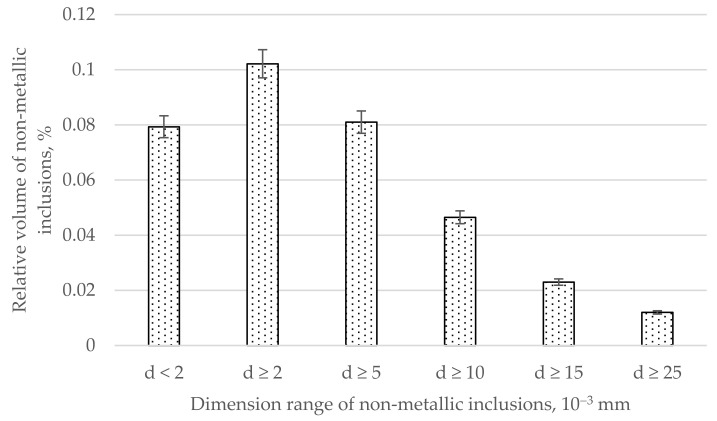
Relative volume of non-metallic inclusions as a dimension range function.

The relative volume of non-metallic inclusions as a dimension range function of the tested steel is shown in Figure 2.

**Figure 3:**
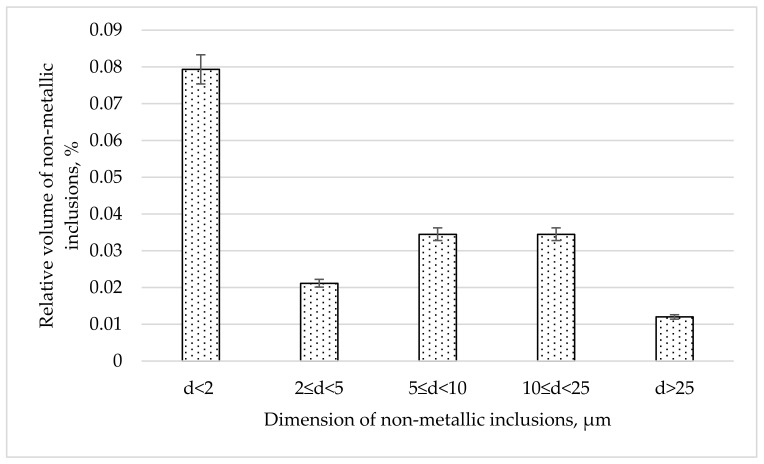
Relative volume of non-metallic inclusions in dimensional ranges.

The analysis of the content of non-metallic inclusions in the analyzed measurement ranges indicates that impurities with diameters below 2 µm constituted 0.079% of the steel volume, those with diameters of 2 µm and larger constituted 0.102%, those with diameters of 5 µm and larger constituted 0.081%, those with diameters of 10 µm and larger constituted 0.047%, those with diameters of 15 µm and larger constituted 0.023%, and those with diameters of 25 µm and larger constituted only 0.012% of the volume of the tested steel (Figure 2). Analyzing the relative volume of non-metallic inclusions in the dimensional ranges (Figure 3), the largest volume of non-metallic inclusions was definitely noted in the range of diameters below 2 µm, amounting to 0.079% of the steel volume. The relative volume in the remaining analyzed dimensional ranges was 0.021% in the range from 2 to <5 µm, 0.035% in the range from 5 to <10 µm and, similarly, in the range from 10 to <25 µm, and 0.012%. for the range of diameters above 25 µm.

**Figure 4:**
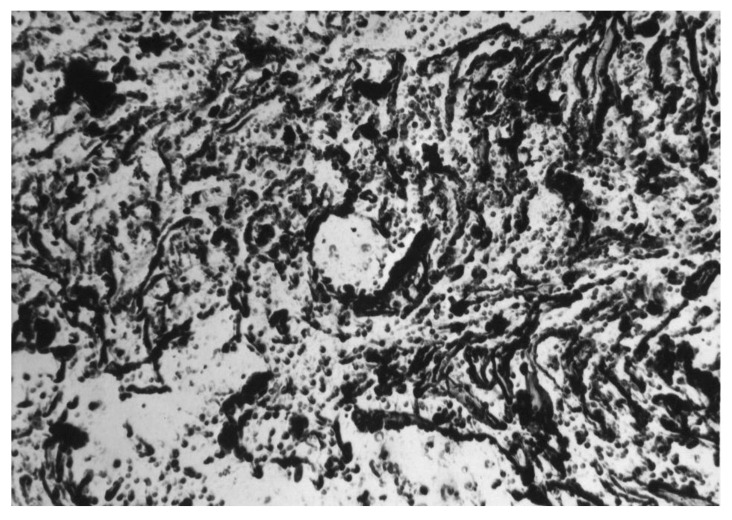
Al_2_O_3_ inclusions in the shape of irregular polyhedrons and small, spherical MgO inclusions.

**Figure 5:**
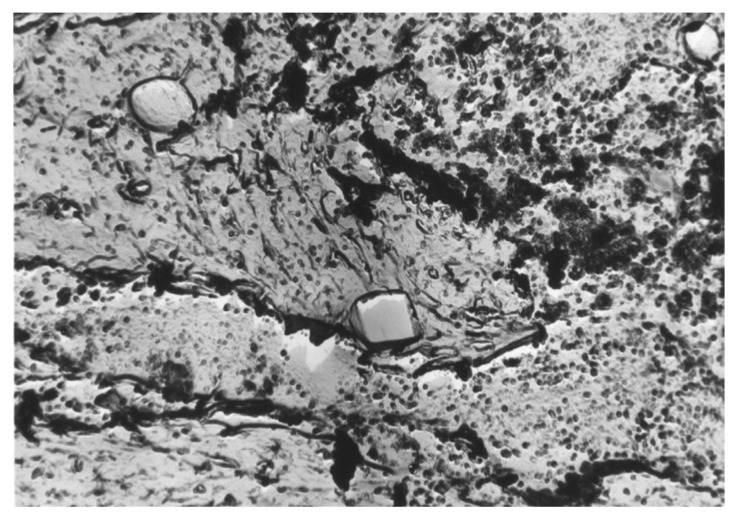
Spherical MgO inclusions and Cr_2_O_3_ parallelepiped inclusions.

Analyzing Figure 2 and Figure 3, it can be seen that the main fraction in the analyzed steel consists of very small non-metallic inclusions with diameters below 2 µm. From this statement in relation to Figure 1, it can be generalized that the dominant fraction is Al_2_O_3_ (Figure 4) with diameters below 2 µm. Taking into account the total volume of all non-metallic inclusions at the level of 0.18% and their decreasing shares with increasing impurities diameters (Figure 2) and shares in individual size ranges of diameters (Figure 3), it can be concluded that the number of non-metallic inclusions in the analyzed steel decreases with the increase in their diameters. Nevertheless, despite all our efforts, it was not possible to eliminate inclusions with diameters larger than 10 µm under the applied industrial conditions. Examples of non-metallic inclusions occurring in the tested steel are presented in Figure 4 and Figure 5.

**Figure 6:**
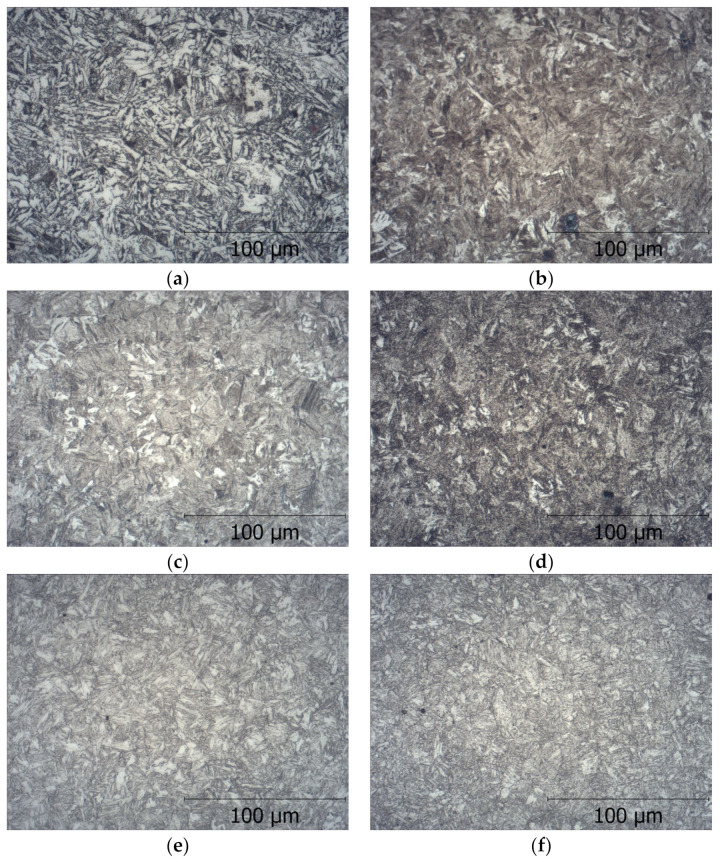
Microstructure of the researched steel when (**a**) hardened at 880 °C without tempered, (**b**) hardened at 880 °C and tempered at 200 °C, (**c**) hardened at 880 °C and tempered at 300 °C, (**d**) hardened at 880 °C and tempered at 400 °C, (**e**) hardened at 880 °C and tempered at 500 °C, and (**f**) hardened at 880 °C and tempered at 600 °C.

The microstructure of austenitizing steel at 880 °C and under tempering at 200, 300, 400, 500, and 600 °C is shown in Figure 6.

The average Vickers hardness and tensile strength of carbon steels hardened and tempered at different temperatures are shown in Table 3.

**Figure 7:**
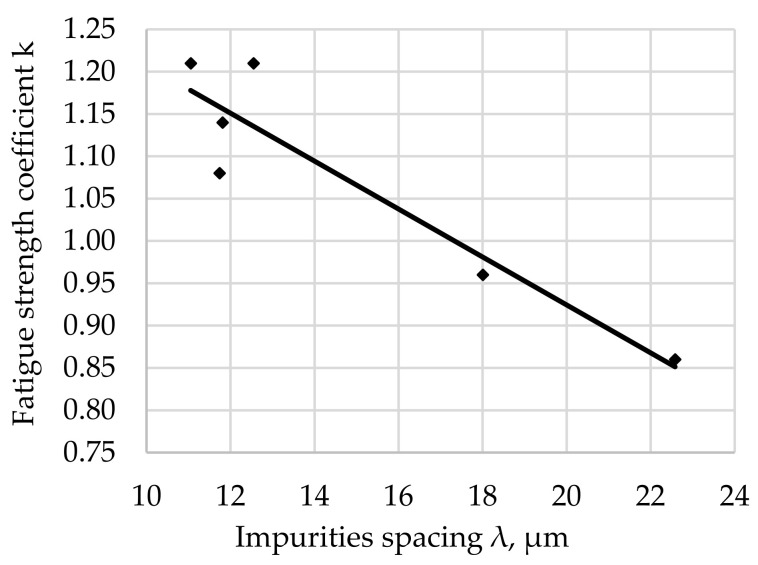
Fatigue resistance coefficient as a function of the impurities spacing λ of structural steel hardened and tempered at 200 °C.

The relationship between the fatigue resistance coefficient and impurities spacing λ for the rotational bending of carbon steel hardened at 880 °C and tempered at 200 °C is shown in Figure 7.

The regression equation with the correlation coefficient r for tested steel tempered at 200 °C is shown as (8).

*k*_(200)_ = −0.0283 · λ + 1.4908 and *r* = 0.9302 (8)

**Figure 8:**
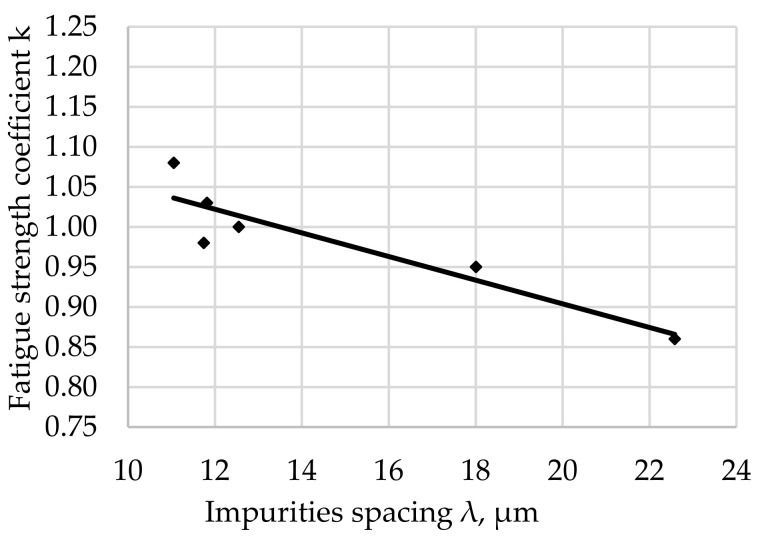
Fatigue resistance coefficient as a function of the impurities spacing λ of structural steel hardened and tempered at 300 °C.

The relationship between the fatigue resistance coefficient and the impurities spacing λ of structural steel hardened at 880 °C and tempered at 300 °C is shown in Figure 8.

The regression equation with the correlation coefficient r for tested steel tempered at 300 °C is shown as (9).

*k*_(300)_ = −0.0148 · λ + 1.1993 and *r* = 0.9152 (9)

**Figure 9:**
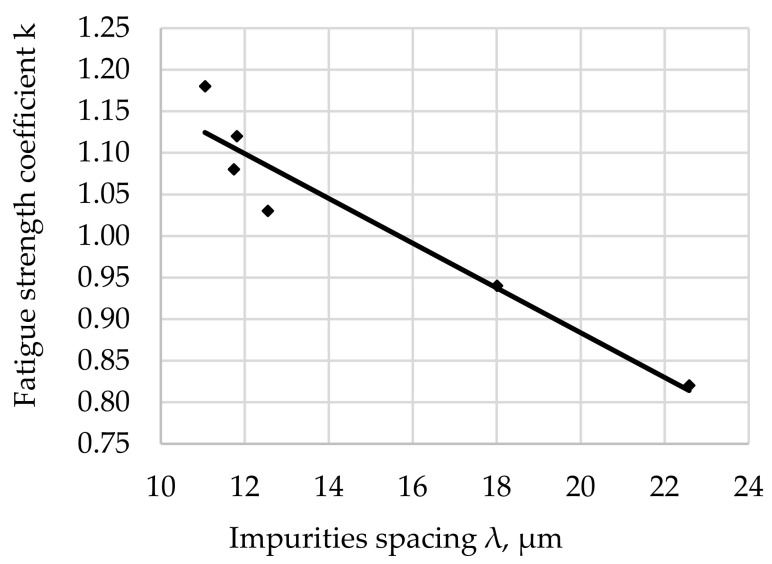
Fatigue resistance coefficient as a function of the impurities spacing λ of structural steel hardened and tempered at 400 °C.

The relationship between the fatigue resistance coefficient and the impurities spacing λ of structural steel hardened at 880 °C and tempered at 400 °C is shown in Figure 9.

The regression equation with the correlation coefficient r for tested steel tempered at 400 °C is shown as (10).

*k*_(400)_ = −0.0269 · λ + 1.422 and *r* = 0.9581 (10)

**Figure 10:**
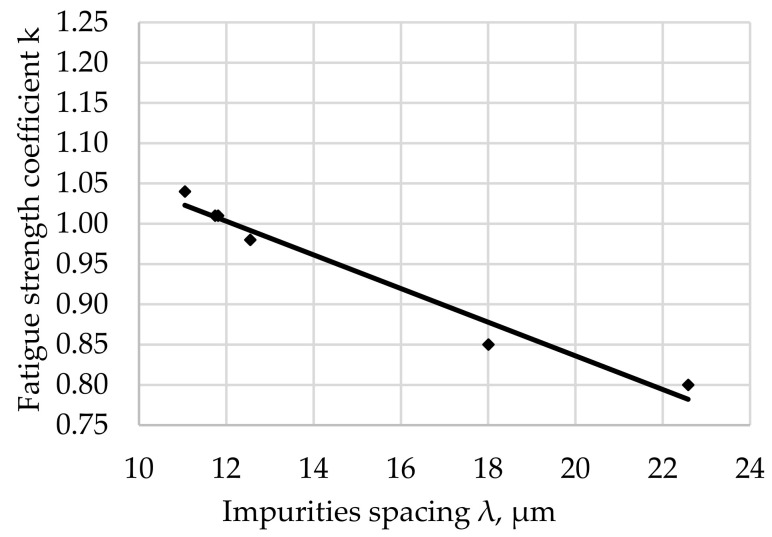
Fatigue resistance coefficient as a function of the impurities spacing λ of structural steel hardened and tempered at 500 °C.

The relationship between the fatigue resistance coefficient and the impurities spacing λ of structural steel hardened at 880 °C and tempered at 500 °C is shown in Figure 10.

The regression equation with the correlation coefficient r for tested steel tempered at 500 °C is shown as (11).

*k*_(500)_ = −0.0209 · λ + 1.2539 and *r* = 0.9780 (11)

**Figure 11:**
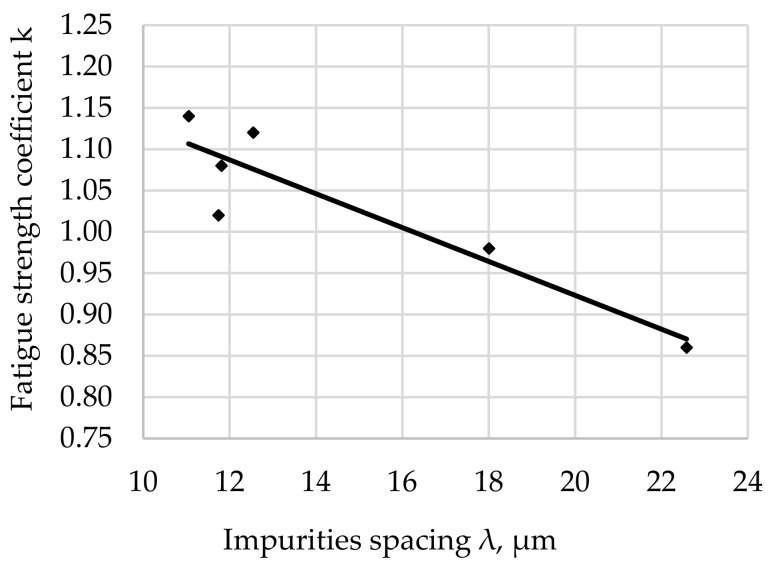
Fatigue resistance coefficient as a function of the impurities spacing λ of structural steel hardened and tempered at 600 °C.

The relationship between the fatigue resistance coefficient and the impurities spacing λ of structural steel hardened at 880 °C and tempered at 600 °C is shown in Figure 11.

The regression equation with the correlation coefficient r for tested steel tempered at 600 °C is shown as (12).

*k*_(600)_ = −0.0205 · λ + 1.3329 and *r* = 0.9152 (12)

**Figure 12:**
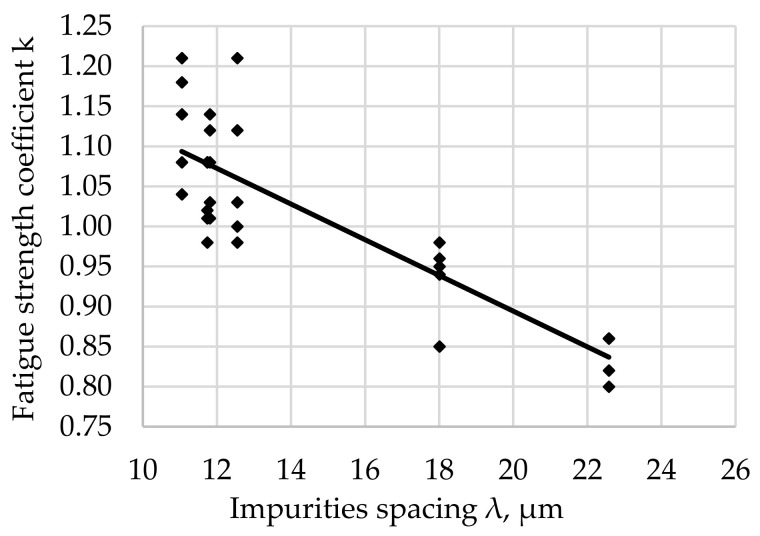
Fatigue resistance coefficient as a function of the impurities spacing λ of structural steel hardened and tempered at all tested temperatures.

The relationship between the fatigue resistance coefficient and the impurities spacing λ of structural steel hardened at 880 °C and tempered at all tested temperatures is shown in Figure 12.

The regression equation with the correlation coefficient r for tested steel tempered at all tested temperatures is shown as (13).

*k*_(all)_ = −0.0223 · λ + 1.3398 and *r* = 0.8456 (13)

The statistical parameters for regression Equations (8)–(13) are presented in Table 4.

## 4. Discussion

The dominant component of non-metallic inclusions is Al_2_O_3_, occupying about 40% of the volume of all impurities (Figure 1). The content of SiO_2_ is almost three times lower and amounts to about 14%. The volume of Cr_2_O_3_, similarly to MgO, MnO, and CaO, was determined to be about 10%. Non-metallic inclusions based on iron oxide were determined to comprise about 8%. Therefore, the dominant component in inclusions is aluminum oxide. This is a hard phase. If it were introduced into the alloy in its existing form, low wettability resulting in a weak connection with the matrix [48] could be expected. However, there is no basis for any conclusion regarding the possibility of introducing Al_2_O_3_ into the melted alloy in the form of the large number of fine particles that we used in our observations over six repetitions of melts. Additionally, in all melts, a comparable content of not only this phase but also other non-metallic inclusions was found. Microscopic observation of the phase boundaries confirmed a good connection between Al_2_O_3_ and the matrix (Figure 4), which indicated the formation of this phase during the metallurgical process. The second phase in terms of the volume occupied in the alloy, in the form of SiO_2_, does not belong to the hard and strong phase like Al_2_O_3_. Silicon oxide, similarly to calcium oxide, is cleavable, with low plasticity [54,55,56,57,58,59,60]. Additionally, it usually occupies large surfaces with irregular shapes, which facilitate their division. Fortunately, the number of precipitations in this phase is not numerous. Precipitations of Cr_2_O_3_ occur in the form of irregular cubes. This phase is characterized by a high degree of fragmentation. It is hard, high-strength, and well bonded with the steel matrix. The corners and sides of this phase are rounded, meaning that it does not constitute sharp notches in the alloy microstructure. Considering that the total content of non-metallic inclusions in the alloy is about 0.18% by volume, this confirms that the tested steel is of high purity, while the distribution of non-metallic inclusions for individual dimensional ranges (Figure 2) confirms that the observed inclusions have small dimensions. The largest number of non-metallic inclusions have a diameter of under 2 µm.

Subjecting the microstructure of steel to hardening and tempering at individual temperatures allowed us to differentiate not only the phase structure (Figure 6) of the steel but also its mechanical properties (Table 3). Steel subjected to hardening and low tempering at a temperature of 200 °C had a microstructure of low-tempered martensite (Figure 6) with a hardness of 409 HV30, and the standard deviation of the results was 57 HV (Table 3). After tempering at each temperature, the following results were obtained: 300 °C—375 HV, 400 °C—345 HV, 500 °C—311 HV, and 600 °C—258 HV

The fatigue strength coefficient k for the tested steel after hardening and degreasing for all the tempering temperatures used indicates an increase in k with an increase in λ. The significance of the effect of λ on the fatigue strength coefficient is illustrated by the slope of the regression line (the angle of inclination to the λ axis). The largest scatter of results illustrated by the correlation coefficient (8) was noted for the tempering temperature of 200 °C (Figure 7). Assuming a comparable morphology and distribution of non-metallic inclusions for all tested samples (such assumptions can be made taking into account the random selection of samples for testing and their large number), it can be concluded that the analyzed fit of the results to the regression curve is a result of the structure of the matrix.

After the steel is tempered at 200 °C, the microstructure is composed of low-tempered martensite, so it is hard, with very low plasticity (the lowest among the tempering temperatures used). Based on the analysis of the literature [61,62,63] and theoretical considerations of the formation and development of cracks in steels [48,64,65], it can be assumed that this matrix may crack as a result of stresses with values higher than permissible or after exceeding the permissible number of cycles for fatigue life. Small-sized non-metallic inclusions of a compact shape (Al_2_O_3_, Cr_2_O_3_) have little effect on fatigue strength [66,67,68,69].

The largest concentration of measurement points in the analyzed Figure 7, Figure 8, Figure 9, Figure 10, Figure 11 and Figure 12 can be found when the range of λ is from 11 to 13 µm. Their arrangement in relation to k indicates the random nature of changes. The exceptions are the results for the tempering temperature of 500 °C (Figure 10), for which all measurement points are arranged in a sequence close to the regression line, which is confirmed by the high correlation coefficient at the level of 0.978. It should be emphasized that it has been determined for six results constituting the arithmetic means of the analyzed population. Analyzing the trends in the changes presented in Figure 7, Figure 8, Figure 9, Figure 10, Figure 11 and Figure 12, it can be seen that, regardless of the tempering temperature representing the proper microstructures and the resulting mechanical properties, the fatigue resistance coefficient k decreases with the increase in impurities spacing. For small impurities spacing of the order of λ < 13 µm, the fatigue strength coefficient is high. Its value depends on the tempering temperature (microstructure). The explanation of this effect will most likely be associated with the effect of small particles on fracture mechanics. Small non-metallic inclusions, striving to maintain minimum energy, usually have oval shapes. This was confirmed by our studies. The comparison of all results (Figure 12) shows that the fatigue strength coefficient value depends on the microstructure represented by the tempering temperature. Assuming a constant volume of non-metallic inclusions in a unit of steel volume, smaller distances between impurities were observed, which confirms the higher density of non-metallic inclusions. With a large share of smaller non-metallic inclusions, a higher fatigue resistance coefficient was noted, which may indicate that small non-metallic inclusions of oval shape do not reduce the fatigue life of steel, regardless of its microstructure.

Research conducted in real conditions also has limitations. The most important include metallographic analysis, i.e., the analysis of steel containing a wide range of non-metallic inclusion dimensions, but in a specific amount (independent of the researcher), and fatigue strength tests, i.e., the randomness of the distribution of impurities.

## 5. Conclusions

Based on the analysis of the test results, we found the following in relation to the tested high-quality steel, produced in large industrial electric furnaces and subjected to desulphurization and argon refining:

- The most numerous inclusions are non-metallic oxide inclusions based on aluminum;
- The volume of non-metallic inclusions decreases exponentially with the increase in their diameter;
- The fatigue resistance coefficient k decreases with the increase in impurities spacing;
- At a low impurities spacing, the tempering temperature, and therefore the microstructure and mechanical properties, has a stronger effect on the fatigue strength coefficient, and it is higher than at higher λ values;
- At a constant volume of non-metallic inclusions, smaller distances between impurities indicate a higher density of non-metallic inclusions;
- At a large share of smaller non-metallic inclusions, a higher fatigue resistance coefficient was noted, which may indicate that small non-metallic inclusions with oval shapes do not reduce the fatigue life of steel, regardless of its microstructure;
- The fatigue strength equations presented here, taking into account the influence of non-metallic inclusions, can be used to develop computer simulation models of fatigue strength in low-carbon, high-purity steel containing impurities. The test results can also be used to predict the strength properties of steel;
- Another interesting area of research seems to be the use of a variable amount of steel scrap and checking how its share in the composition can affect the purity of the steel.

## Figures and Tables

**Table 1:** Average contents of the chemical composition of the tested steel, with standard deviation.

Chemical Element	C wt. %	Mn wt. %	P wt. %	S wt. %	Cr wt. %	Mo wt. %	Ni wt. %	Si wt. %	Cu wt. %	B wt. %
Average contents	0.23	1.23	0.022	0.011	0.47	0.23	0.500	0.236	0.15	0.0025
Standard deviation	0.018	0.128	0.0041	0.0037	0.063	0.016	0.040	0.069	0.012	0.00084

**Table 2:** Applied load and sample size for the fatigue strength test at the assumed tempering temperatures.

Tempering Temperature	°C	200	300	400	500	600
Applied load	MPa	650	600	600	600	540
Sample size	Amount	55	62	60	63	61

**Table 3:** Average Vickers hardness and tensile strength for the tested tempering temperature.

Tempering Temperature	°C	200	300	400	500	600
Vickers hardness	HV	409	375	345	311	258
Standard deviation	HV	57	36	31	10	20
Tensile strength	MPa	1335	1198	1119	997	836
Standard deviation	MPa	39	37	35	34	31

**Table 4:** Dissipation fatigue resistance coefficients, correlation coefficients, and test probability.

Tempering Temperature °C	Dissipation Fatigue Resistance Coefficient *k* (6)	Correlation Coefficient *r*	Test Probability *t_α_* = 0.05	Critical Value from Student’s Distribution for *t_α_* = 0.05 and *p* = (*n* − 1)
200	0.1067	0.9302	6.2076	
300	0.0584	0.9152	5.5627	
400	0.0877	0.9581	8.1934	2.4469
500	0.0426	0.9780	11.4840	
600	0.0797	0.9152	5.5627	
all	0.1237	0.8456	9.2366	2.0452

## Data Availability

The original contributions presented in the study are included in the article, further inquiries can be directed to the corresponding author.
